# Synergistic Cardiac Protection by Thymoquinone and Valproic Acid in Absence Epilepsy

**DOI:** 10.33549/physiolres.935733

**Published:** 2026-04-01

**Authors:** Yasin Ali CIMEN, Fatma Bedia KARAKAYA-CIMEN, Huri DEMIRCI, Ozlem Tugce CILINGIR-KAYA

**Affiliations:** 1 Department of Physiology, Faculty of Medicine, Yalova University, Yalova, Turkey; 2 Department of Physiology, Faculty of Medicine, Bezmialem Vakif University, Istanbul, Turkey; 3 Department of Histology and Embryology, Faculty of Medicine, Yalova University, Yalova, Turkey; 4 Department of Histology and Embryology, Faculty of Medicine, Marmara University, Istanbul, Turkey; 5 Department of Histology and Embryology, Faculty of Medicine, Bezmialem Vakif University, Istanbul, Turkey; 6 Department of Medical Biochemistry, Faculty of Medicine, Biruni University, Istanbul, Turkey; 7 Biruni University Research Center (Bamer), Biruni University, Istanbul, Turkey

**Keywords:** Absence epilepsy, Apoptosis, Inflammation, Lipid peroxidation, Oxidative stress

## Abstract

This study aimed to investigate the effects of thymoquinone (T) and valproic acid (VPA), alone and in combination, on cardiomyocyte damage induced by absence epilepsy (AE) in rats. Adult male Wistar rats (C, T) and Genetic Absence Epilepsy Rats from Strasbourg (GAERS; AE, AE+T, AE+V, AE+V+T) were assigned to six experimental groups. C and AE received solvent; T and AE+T received thymoquinone (10 mg/kg, o.g.); AE+V received VPA (200 mg/kg, i.p.); AE+V+T received both treatments for 8 days. Hearts were collected for histological (Masson’s trichrome) and biochemical analyses. AE caused molecular changes in cardiomyocytes without fibrosis, decreasing total antioxidant status (TAS) (−8.7 %) and increasing total oxidant status (TOS) (+50.7 %), oxidative stress index (OSI) (+155.5 %), MDA (+50.8 %), caspase-8 (+43.8 %), cleaved caspase-3 (+73.5 %), NF-κB (+239.4 %), TNF-α (+141.2 %), IL-6 (+12.9 %). Compared with the AE group, T increased TAS (+48.2 %), IL-6 (+5.8 %) and reduced OSI (−36.7 %), caspase-8 (−16.0 %), cleaved caspase-3 (−37.5 %), TNF-α (−1.8 %). VPA increased TAS (+58.5 %), while reducing TOS (−15.7 %), OSI (−48.9 %), MDA (−31.9 %), IL-6 (−14.1 %), caspase-8 (−27.3 %), cleaved caspase-3 (−51.0 %), TNF-α (−8.9 %), and NF﷓κB (−28.5 %). The VPA+T combination further improved these alterations, decreasing TOS (−24.8 %), OSI (−54.5 %), MDA (−35.6 %), TNF-α (−16.3 %), caspase-8 (−27.7 %), cleaved caspase-3 (−47.1 %), NF-κB (−31.8 %) and IL-6 (−26.9 %) while increasing TAS (+59.8 %). Our study demonstrates that AE increases oxidative stress, apoptosis and inflammation in cardiac tissue. While T and VPA alone showed limited protective effects, their combined administration significantly attenuated cardiac damage, supporting this approach as a promising strategy for cardiac protection.

## Graphical Abstract

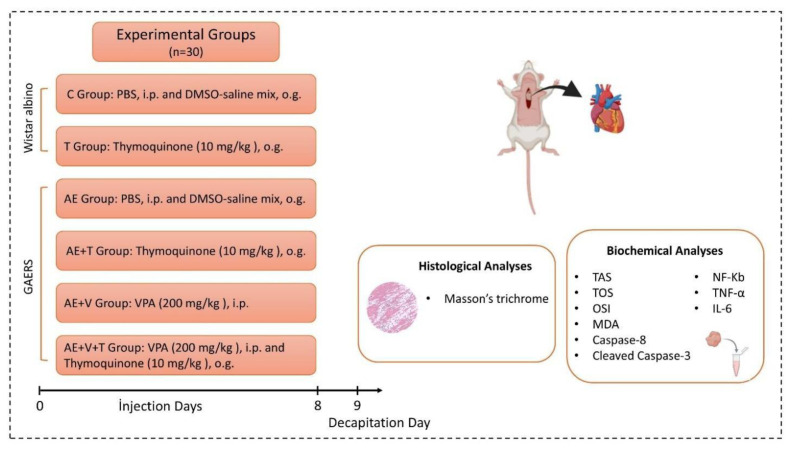

## Introduction

Epilepsy is a common neurological disorder that affects approximately 70 million individuals worldwide [1]. Epilepsy is divided into four main categories according to seizure onset: focal onset, generalized onset, combined generalized onset, and unknown onset [2]. Absence epilepsy (AE) is a type of generalized epilepsy characterized by non-convulsive seizures, sudden cessation of behavior, and transient impairment of consciousness [3]. One of the most common causes of death in epilepsy is sudden unexpected death (SUDEP), which occurs especially in patients with frequent and uncontrolled seizures [4]. Various conditions such as structural heart changes that may result from previous seizure activity, and autonomic changes due to antiepileptic drug exposure may increase the risk of SUDEP [5]. Recent reports indicate that epilepsy is a frequent cause of death in patients and that epilepsy increases the risk of hypertension, coronary artery disease, and myocardial infarction [6].

Valproic acid (VPA), one of the most commonly used anti-epileptic medications, has been reported to be effective in managing absence seizures in 75 % of children [7]. However, in 20 % of epilepsy cases, seizure control cannot be achieved with current medications, and the risk of seizure recurrence persists [8]. Therefore, numerous studies have explored alternative treatments for epilepsy and therapies that can be used alongside traditional anti-epileptic drugs. Animal models of seizures and epilepsy have played a fundamental role in understanding the mechanisms of epileptogenesis and in developing new antiepileptic drugs [9]. The Genetic Absence Epilepsy Rats from Strasbourg (GAERS) model is a reliable animal model that reflects AE and is frequently used in epilepsy research because it exhibits spontaneous absence seizures [10]. Recent research on volatile oils and their primary components has garnered significant interest from scientists, prompting them to investigate these natural products [11]. Notably, some of these plants have demonstrated anti-epileptic effects and have a long history of use in traditional medicine [12]. Nigella sativa is a plant belonging to the Ranunculaceae family and is widely used in both modern medicine and complementary/alternative applications. The main biologically active component in the essential oil of this plant is thymoquinone (C_10_H_12_O_2_; 2-isopropyl-5-methyl-1,4-benzoquinone) [13]. Studies have shown cardio-protective, hepatoprotective, neuroprotective and antiepi-leptic effects [14] of thymoquinone. Furthermore, numerous studies have indicated that thymoquinone has no side effects and does not pose serious toxicity risk [15]. In line with these findings, the safety profile and therapeutic potential of thymoquinone make it possible to use it as a complementary agent with existing antiepileptic drugs in the treatment of epilepsy.

Although the underlying mechanisms of epilepsy-related cardiac damage are increasingly being investigated, the direct effects of antiepileptic drugs on cardiac tissue are limited. Most previous cardiac-epilepsy studies have focused on temporal lobe epilepsy, leaving a gap in the knowledge regarding AE. In this study, GAERS, a validated model of human AE characterized by spontaneous absence seizures from early life and shown to affect autonomic and cardiovascular regulation, was used. We hypothesized that combining antioxidant thymoquinone with VPA may provide additive or synergistic protection against AE-induced cardiac damage. Therefore, the study aimed to investigate the effects of thymoquinone and VPA, alone or in combination, on AE- related cardiac damage in GAERS and Wistar rats.

## Methods

### Animals

A total of thirty male rats (ten Wistar and twenty GAERS, 3 months old, weighing approximately 300 g) were included in the experiment. The animals were housed in cages in air-conditioned rooms at 21 °C with a 12-h light/dark cycle with access to food and water. The experimental protocol was approved by the Experimental Animal Ethics Committee of Marmara University (02.2025mar.6).

### Study design and treatments

The study design is illustrated in the Graphical abstract. The experimental animals were divided into six groups. Wistar rats were assigned to the Control (C) and Thymoquinone (T) groups; GAERS rats were assigned to four groups related to the absence epilepsy model. The six groups were formed as follows:

1- Control (C, Wistar, n=5): Animals received PBS intraperitoneally (i.p.) and corn oil orally (o.g.) for 8 days.
2- Thymoquinone (T, Wistar, n=5): Animals received 10 mg/kg thymoquinone dissolved in corn oil orally for 8 days.
3- Absence Epilepsy (AE, GAERS, n=5): GAERS rats in this group received PBS i.p. and corn oil o.g. for 8 days.
4- Absence Epilepsy + Thymoquinone (AE+T, GAERS, n=5): Animals received 10 mg/kg thymoquinone dissolved in corn oil orally for 8 days.
5- Absence Epilepsy + Valproic Acid (AE+V, GAERS, n=5): Animals received 200 mg/kg VPA dissolved in PBS i.p. for 8 days.
6- Absence Epilepsy + Valproic Acid + Thymoquinone (AE+V+T, GAERS, n=5): Animals received both 200 mg/kg VPA i.p. and 10 mg/kg thymoquinone orally for 8 days.

Animals were sacrificed under anesthesia 24 hours after the final injection. The cardiac tissue was carefully dissected. Half of the cardiac tissue was immersed in 10 % neutral buffered formalin, while the other half was placed in a −80 °C freezer for further analyses using western blotting and ELISA.

### Morphologic analysis of cardiac tissue

The samples were fixed in 10 % neutral buffered formalin and dehydrated using a series of ascending alcohol solutions (90 %, 96 %, and 100 %). Following dehydration, the samples were cleared in xylene until they became transparent, after which they were immersed in liquid paraffin and subsequently embedded using a paraffin-blocking device (EG1150H; Leica, Germany). Sections of 4 μm thickness were cut from the paraffin blocks and stained with Masson’s trichrome staining was performed to highlight changes in the connective tissue [16]. Cardiac tissue images were captured using a Nikon light microscope (Eclipse 920248, USA) at x40 magnification. Histological evaluation was performed blinded to treatment groups.

### Tissue homogenization

At sacrifice, half of the cardiac tissues were removed to a −80 °C refrigerator were washed in 0.9 % NaCl. The samples were then homogenized in a cold buffer (1.15 % KCl containing 100 mM KH_2_PO_4_-K_2_HPO_4_; pH 7.4) using a homogenizer (Merck Millipore, USA). All samples were centrifuged at 2500x g for 10 min just before analysis and the liquid component was transferred to a new Eppendorf tube.

### Western Blot Analysis

To minimize inter-individual variability and to obtain a representative protein profile for each experimental group, cardiac tissue homogenates from all animals within the same group were pooled in equal amounts prior to Western Blot analysis. The pooled homogenates were subsequently processed for protein extraction and immunoblotting. For Western Blot analysis, separated tissue samples were kept on ice for 30 minutes in a pre-prepared RIPA cell lysis buffer containing a protease inhibitor cocktail. The samples were then centrifuged at 13,000 rpm for 10 minutes at 4 °C, and the resulting supernatant was used as the cytosolic fraction. Total protein concentrations were determined using the Bradford protein assay [17]. Proteins were separated on an 8–12 % SDS-PAGE gel and subsequently transferred onto a PVDF membrane.

Following transfer, membranes were blocked with 5 % non-fat dry milk in TBS-T and then incubated overnight at 4 °C with the respective primary antibodies, including anti–caspase-8 (Elabscience, USA), anti–cleaved caspase-3 (Affinity Bioscience, China), anti–NF-κB (Affinity Bioscience, China), anti–TNF-α (Elabscience, USA), anti–IL-6 (Affinity Bioscience, China), and anti–β-actin. After primary antibody incubation, membranes were washed and incubated with the appropriate horseradish peroxidase (HRP)-conjugated secondary antibodies.

Protein bands were visualized using Pierce ECL WB substrate (Bio-Rad, CA, USA). The bands formed for each antibody were imaged with a Vilber Lourmat Fusion Fx5 imaging system. Band intensities were quantified using ImageJ software (National Institutes of Health, Bethesda, MD, USA). Densitometric measurements were performed on non-saturated bands, and the intensity of each target protein was normalized to the corresponding β-actin band.

### Biochemical analyses

Biochemical analyses were performed on heart tissue homogenates isolated from the animals. Proprietary ELISA kits were used to test malondialdehyde (MDA), which indicates lipid peroxidation (BT-LAB-E0156Ra, Shanghai, China). Analyses were performed according to the manufacturer’s protocols. The total oxidant status (TOS) and total antioxidant status (TAS) were determined from hearts using proprietary kits (Rel Assay Diagnostics, Gaziantep, Turkey) according to the manufacturer’s instructions. The oxidative stress index [18] was calculated from the TOS and TAS levels as follows:


$$\text{OSI}=[\text{TOS }(\mu \text{mol }{\text{H}}_{2}{\text{O}}_{2}\;\text{equi.}/\text{l})/\text{TAS }(\mu \text{mol trolox equi.}/\text{l})]\times 100$$

### Statistical analysis

In the power analysis performed, it was determined that the study was conducted with a 95 % confidence level (α=0.05) using a total of 30 animals in 6 groups with n=5 per group, and that the effect obtained could be detected with 88.82 % power (power=0.8882). The calculated effect size is 0.8158. Power analysis was performed using the MDA parameter, and all calculations were performed using PASS 2008 [19]. The data obtained from the study were analyzed using GraphPad Prism 8 (GraphPad Software, San Diego, CA, USA). Data are presented as mean ± standard deviation (mean ± SD). Normal distribution was assessed using the Shapiro–Wilk test. Homogeneity of variance was verified using the Brown–Forsythe and Bartlett tests, which were automatically applied by GraphPad Prism. After these assumptions were met, one-way ANOVA was performed, and comparisons between groups were made using Tukey’s multiple comparison test. p < 0.05 was considered statistically significant. Schematics were generated using BioRender.com.

## Results

### Histological results

**Fig. 1:**
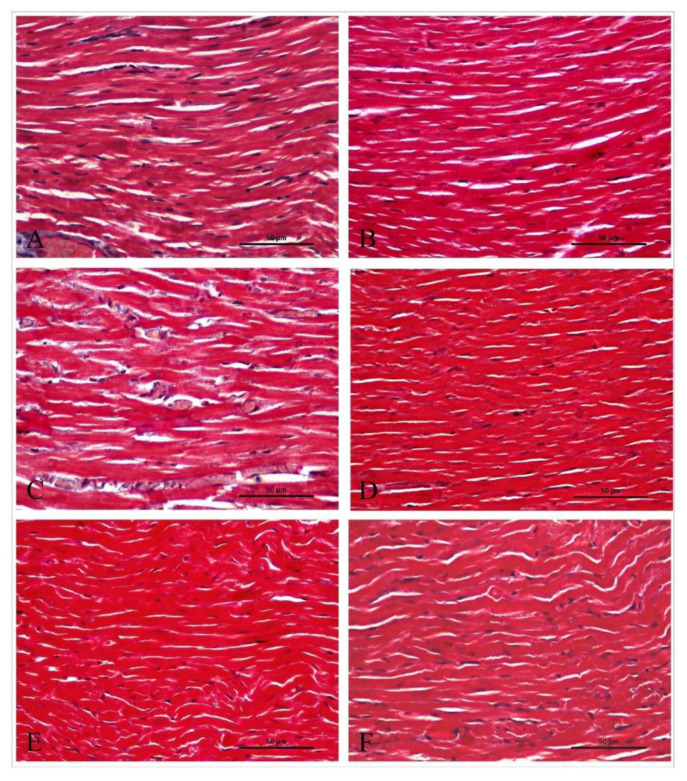
Representative Masson’s trichrome–stained sections from the experimental groups. **A**) C group, **B**) T group, **C**) AE group, **D**) AE+T group, **E**) AE+V group, **F**) AE+V+T group. (mag: x40, n=5 per group).

Masson’s trichrome staining method was applied to evaluate the structural changes in the connective tissue and possible fibrosis in more detail. Masson’s trichrome staining results showed no significant difference between the groups in terms of collagen fiber density and distribution (Fig. 1 A–F).

### Oxidative stress results

**Fig. 2:**
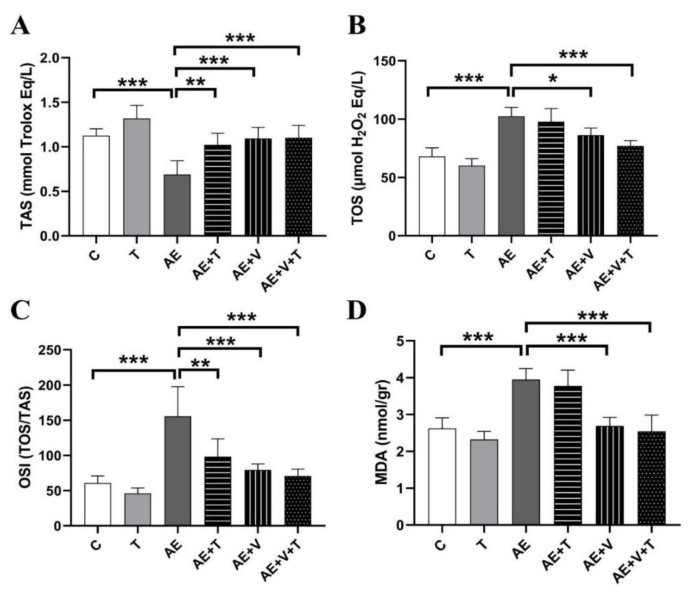
**A**) Total Antioxidant Status (TAS), **B**) Total Oxidant Status (TOS), **C**) Oxidative Stress Index (OSI) and **D**) Malondialdehyde (MDA) levels in the cardiac tissues of experimental groups (n=5 per group). *p<0.05, **p<0.01, ***p<0.001 indicating levels of statistical significance. C, control; T, thymoquinone; AE, absence epilepsy; AE+T, absence epilepsy treated with thymoquinone; AE+V, absence epilepsy treated with valproic acid; AE+V+T, absence epilepsy treated with valproic acid and thymoquinone.

No significant differences were observed between the T and control groups in oxidative stress parameters. TAS, TOS, OSI and MDA levels were measured to evaluate the effects of epilepsy, thymoquinone and VPA-induced oxidative damage in cardiac tissue to reveal the presence and severity of oxidative damage. TAS value decreased significantly in the AE group compared to that in the C group (p<0.001). In the AE+T (p<0.01), AE+V (p<0.001), and AE+V+T (p<0.001) groups, the decreased TAS levels in the GS group reached levels similar to those of the controls (Fig. 2A).

TOS value increased in the AE group compared to that in the C group (p<0.001). TOS value decreased significantly in the AE+V (p<0.05) and AE+V+T (p<0.001) groups (Fig. 2B).

While OSI levels in the AE group increased significantly compared to the C group (p<0.001), they decreased significantly in the AE+T (P<0.01), AE+V (P<0.001) and AE+V+T (p<0.001) groups compared to the AE group (Fig. 2C).

While MDA level increased significantly in the AE (p<0.001) group compared to the C group, it decreased significantly in the AE+V (P<0.001) and AE+V+T (p<0.001) groups (Fig. 2D).

### Apoptosis results

**Fig. 3:**
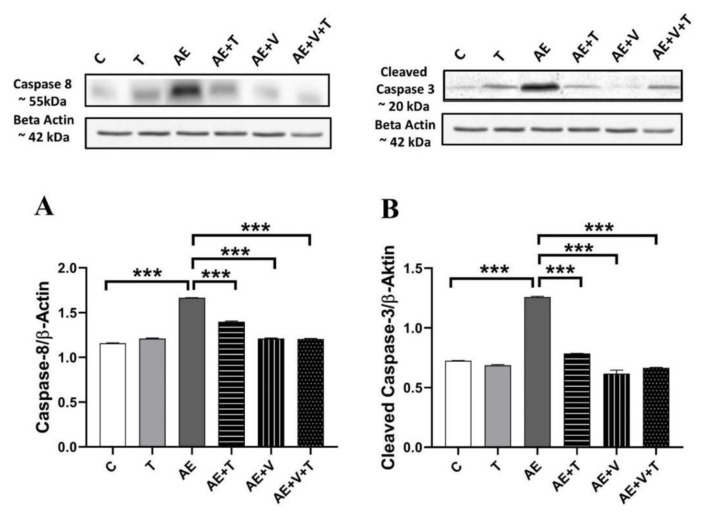
Caspase-8 (**A**) and cleaved Caspase-3 (**B**) expres-sions in the cardiac tissues of experimental groups (n=5 per group). *p<0.05, **p<0.01, ***p<0.001 indicating levels of statistical significance. For abbreviation see Fig. 2.

No significant differences were observed between the T and control groups in apoptosis parameters. Caspase-8 and Cleaved Caspase-3 levels were measured to evaluate the activation of apoptotic pathways mediating cell death that may occur in epilepsy, thymoquinone and VPA-induced cardiac tissue. Caspase-8 and cleaved Caspase-3 levels increased significantly in the AE group compared to the C group (p<0.001), they significantly decreased in the AE+T (P<0.001), AE+V (P<0.001) and AE+V+T (P<0.001) groups (Fig. 3A, B).

### Inflammation marker results

**Fig. 4:**
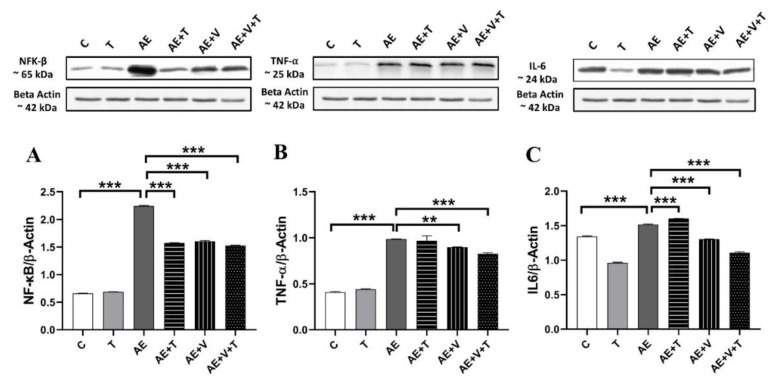
NF-κB, TNF-α, and IL-6 (**A–C**) expressions in the cardiac tissues of experimental groups (n=5 per group). *p<0.05, **p<0.01, ***p<0.001 indicating levels of statistical significance. For abbreviation see Fig. 2.

No significant differences were observed between the T and control groups in inflammation parameters. We evaluated NF-κB, TNF-α and IL-6 levels to assess the severity of inflammation and possible pathological changes in cardiac tissue due to epilepsy, thymoquinone and VPA. NF-κB expression significantly increased in the AE group and decreased in the AE+T, AE+V, and AE+V+T groups (p<0.001; Fig. 4A). TNF-α expression increased significantly in group AE (p<0.001) and decreased in AE+V (p<0.01), AE+V+T (p<0.001) groups (Fig. 4B). IL-6 expression significantly increased in the AE and AE+T groups and significantly decreased in the AE+V and AE+V+T groups (p<0.001) (Fig. 4C).

## Discussion

In this study, the effects of thymoquinone, VPA, and their combinations on AE-related heart damage were evaluated based on histological examination, oxidative stress, apoptosis, and inflammation parameters. This study is the first to examine the effects of TQ alone or in combination with VPA on heart damage caused by AE.

It has been reported that changes in cardiac structure and function in patients with epilepsy may lead to fatal tachycardia and SUDEP risk [20]. In this context, no fibrotic changes were detected in the sections prepared with Masson’s trichrome staining in our study. The lack of significant histological changes may be related to the shorter seizure duration and less aggressive course of absence epilepsy compared to other types of epilepsy. Indeed, cardiac hypertrophy has been reported in the temporal lobe epilepsy model induced by kainic acid, but no fibrotic tissue was observed, which is consistent with our findings [21]. However, some studies have reported that epileptic seizures cause cardiac fibrosis in temporal lobe epilepsy models [22]. This situation indicates that the effects of epilepsy on heart structure and function need to be investigated more comprehensively using different animal models and conditions.

Increased neuronal activity during epileptic seizures can disrupt mitochondrial metabolism by increasing oxygen consumption and lead to excessive production of reactive oxygen species [23]. Mitochondrial dysfunction has been directly associated with epilepsy in humans [24]. The harmful effects of increased free radicals in epileptic seizures are felt not only in the brain but also in the heart, particularly due to its high energy requirements [25]. Moreover, energy production is reduced due to mitochondrial damage, which can trigger pathological processes such as apoptosis and fibrosis [26]. These observations indicate the need for more detailed research into the effects of oxidative stress caused by epilepsy on the heart. In our study, we found that TAS decreased and TOS, OSI and MDA levels increased in the hearts of rats with AE. In addition, we found a decreasing trend in TOS and MDA levels, an increase in TAS levels, and a significant decrease in OSI levels with thymoquinone administration. These findings are consistent with the antioxidant effects of thymoquinone reported in the literature; it has been shown to increase decreased serum TAS levels and decrease increased TOS levels in a pilocarpine-induced epilepsy model [27], to decrease TOS levels and improve TAS levels in doxorubicin-induced cardiotoxicity [28], and to decrease MDA levels in isoproterenol-induced myocardial damage [29]. Our findings and the present data suggest that thymoquinone may be a potential protective agent in reducing oxidative stress in epilepsy-induced cardiac damage. Following VPA treatment, MDA levels were observed to decrease in animals in the AE group. Tastemur and colleagues also administered the VPA dose used in our study to epileptic animals and obtained similar results [30]. When we investigated the simultaneous use of thymoquinone with VPA, we observed an increase in TAS levels and a decrease in TOS, OSI, and MDA levels. These findings suggest that the simultaneous use of VPA and thymoquinone may be beneficial in reducing oxidative stress and lipid peroxidation caused by AE.

Studies investigating the relationship between epilepsy and apoptosis have shown that Bcl-2 levels are reduced in cardiomyocytes of genetically epileptic rats, while BAX and caspase-3 expression and the number of TUNEL-positive cells are increased [31]. Subsequent research has further supported this relationship with increased norepinephrine levels, prolonged QT intervals, histological changes, and proliferation of apoptotic cardiomyocytes in epileptic animals [32]. Similarly, in our study, caspase-8 and cleaved caspase-3 levels were increased in animals with AE, suggesting that epilepsy triggers apoptosis in cardiac tissue. There is no study in the literature directly demonstrating the effect of thymoquinone on epilepsy-induced apoptotic damage in cardiac tissue; however, it has been reported that TQ increases Bcl-2 expression and decreases caspase-3 activity in the cortex and hippocampus of epileptic animals [33]. Additionally, it has been reported that TQ alleviates myocardial ischemia/reperfusion injury and reduces mitochondrial oxidative stress and cardiomyocyte apoptosis [34]. In the present study, we confirmed the anti-apoptotic effect of thymoquinone by reducing caspase-8 and cleaved caspase-3 levels. The VPA application alone prevented apoptosis by reducing the levels of increased caspase-8 and cleaved caspase-3 in AE-induced cardiac tissue. Studies directly examining the effect of VPA on cardiac apoptosis in AE are limited; however, it is known that VPA prevents myocardial cell damage by reducing oxidative stress and inflammation, thereby preserving cardiac function and tissue integrity [35, 36]. Additionally, in our study, the simultaneous administration of TQ and VPA similarly reduced these apoptotic markers, thereby more effectively inhibiting apoptosis and demonstrating a potential synergistic protective effect.

The role of cytokines in the development of epileptic seizures is increasingly recognized. Following seizures, it has been demonstrated in experimental models and clinical studies that proinflammatory cytokines in the brain are rapidly activated and trigger inflammatory responses [37]. Cytokines such as IL-1β, IL-2, IL-6, and TNF-α are found at low levels in the healthy brain, but increase significantly after ischemic, traumatic, or excitotoxic damage and generalized seizures [38]. These cytokines, TNF-α, TGF-β, IL-1, IL-4, and IL-6, play a critical role in the development of inflammatory heart diseases such as ischemic heart disease, myocardial infarction, heart failure, and cardiomyopathies and can affect cardiac function via nuclear transcription factors such as NF-κB [39]. Therefore, it is important to consider these inflammatory factors when examining cardiac parameters in animal models treated with antiepileptic drugs. Although studies directly examining cardiac inflammation in epilepsy are limited, increased levels of TNF-α, NF-κB, and IL-6 have been reported in doxorubicin-induced cardiac injury models [40]. In our study, we found that TNF-α, NF-κB, and IL-6 levels were increased in the hearts of animals with AE. The administration of thymoquinone alone reduced NF-κB levels but did not affect TNF-α and led to an increase in IL-6 levels. Although the anti-inflammatory cardiac effects of thymoquinone have not been directly investigated in epileptic models, current data indicate that this compound has a significant anti-inflammatory potential. In seizure models, thymoquinone reduced TNF-α and IL-6 levels [41], showed protective effects by modulating the NF-κB signaling pathway in pilocarpine-induced brain injury [42]. Thymoquinone has been reported to reduce inflammation by decreasing TNF-α and IL-6 levels in cardiac injury [43]. In our study, an increase in IL-6 levels was observed; this is thought to be explained by IL-6’s ability to exhibit both proinflammatory and anti-inflammatory effects [37, 44]. Similarly, in our study, VPA administration demonstrated significant anti-inflammatory effects by reducing TNF-α, NF-κB, and IL-6 levels. Reports in the literature that VPA treatment is associated with NF-κB inhibition and decreased expression of IL-1β, TNF-α, and IL-6 in animal models support our findings [45]. In VPA-treated groups, the suppression of caspase-3 observed in conjunction with NF-κB inhibition suggests that the attenuation of inflammation may involve oxidative stress–mediated apoptotic pathways. In the group where both VPA and thymoquinone were administered together, although NF-κB levels remained at similar levels to those seen with the agents administered alone, a significant decrease in TNF-α and IL-6 levels was observed. These results demonstrate that the simultaneous use of VPA and thymoquinone suppresses the inflammatory response more effectively than the administration of either agent alone.

## Conclusions

This study comprehensively demonstrates for the first time the oxidative stress, apoptosis, and inflammatory damage caused by epilepsy in cardiac tissue within the AE model framework. The findings reveal the therapeutic potential of thymoquinone and VPA in modulating this damage and emphasize that the combination of both agents provides stronger protective effects compared to treatments administered alone. The results obtained reveal that epilepsy is not limited to the central nervous system but also causes systemic effects in cardiac tissue, highlighting the importance of evaluating these effects with multidisciplinary approaches. Furthermore, these findings provide a valuable foundation that may guide clinical applications in the prevention or treatment of AE-induced cardiac damage in the future.

## Limitations

This study has certain limitations. The relatively small sample size and 8-day treatment period limit the generalizability of the results and the assessment of long-term effects. The use of only adult male rats did not allow for the investigation of possible gender differences. Furthermore, since cardiac functions were not evaluated, the findings are limited to biochemical and histological data.
